# Tubulin-VDAC Interaction: Molecular Basis for Mitochondrial Dysfunction in Chemotherapy-Induced Peripheral Neuropathy

**DOI:** 10.3389/fphys.2019.00671

**Published:** 2019-05-31

**Authors:** Amandine Rovini

**Affiliations:** Department of Drug Discovery and Biomedical Sciences, Medical University of South Carolina, Charleston, SC, United States

**Keywords:** tubulin, VDAC, mitochondria, neurotoxicity, chemotherapy

## Abstract

Tubulin is a well-established target of microtubule-targeting agents (MTAs), a widely used class of chemotherapeutic drugs. Yet, aside from their powerful anti-cancer efficiency, MTAs induce a dose-limiting and debilitating peripheral neurotoxicity. Despite intensive efforts in the development of neuroprotective agents, there are currently no approved therapies to effectively manage chemotherapy-induced peripheral neuropathy (CIPN). Over the last decade, attempts to unravel the pathomechanisms underlying the development of CIPN led to the observation that mitochondrial dysfunctions stand as a common feature associated with axonal degeneration. Concomitantly, mitochondria emerged as crucial players in the anti-cancer efficiency of MTAs. The findings that free dimeric tubulin could be associated with mitochondrial membranes and interact directly with the voltage-dependent anion channels (VDACs) located in the mitochondrial outer membrane strongly suggested the existence of an interplay between both subcellular compartments. The biological relevance of the interaction between tubulin and VDAC came from subsequent *in vitro* studies, which found dimeric tubulin to be a potent modulator of VDAC and ultimately of mitochondrial membrane permeability to respiratory substrates. Therefore, one of the hypothetic mechanisms of CIPN implies that MTAs, by binding directly to the tubulin associated with VDAC, interferes with mitochondrial function in the peripheral nervous system. We review here the foundations of this hypothesis and discuss them in light of the current knowledge. A focus is set on the molecular mechanisms behind MTA interference with dimeric tubulin and VDAC interaction, the potential relevance of tubulin isotypes and availability as a free dimer in the specific context of MTA-induced CIPN. We further highlight the emerging interest for VDAC and its interacting partners as a promising therapeutic target in neurodegeneration.

## Introduction

Most of the chemotherapeutic agents in clinical use currently exhibit a dose limiting neurological toxicity, irrespective of their class. Damages to peripheral nerves, referred to as chemotherapy-induced peripheral neuropathy (CIPN), constitute a major cause of dose reduction and treatment interruption in patients with cancer. These side effects can last months to years following treatment and can be extremely challenging to manage on a daily basis for patients and cancer survivors. The prevalence and reversal of CIPN vary depending on the type of agent, dose per cure, and cumulated dose as well as co-administration of other agents (Staff et al., 2017; Starobova and Vetter, 2017). CIPNs are primarily caused by the development of axonopathy (through dying back axonal damage) and neuronopathy (with the involvement of the dorsal root ganglia), but the precise pathophysiology is not clearly understood. Similarly, the contribution of non-neuronal cells, such as Schwann cells to CIPN, is not fully elucidated (Cavaletti, 2014; Seretny et al., 2014; Fehrenbacher, 2015; Kim et al., 2015). Unfortunately, a major clinical drawback is the current lack of preventive strategies and limited FDA approved treatment options to provide symptomatic relief. Therefore, since management of CIPN patients remains an unmet clinical need, extensive efforts have been dedicated to target the source by exploring, in pre-clinical and *in vitro* models, the molecular mechanisms at play in the etiology of CIPN (Ma et al., 2018). Multiple mechanisms have been proposed to underlie or to correlate with the emergence of CIPN including inflammatory processes, ion channel disturbance, alteration of microtubule dynamics, and oxidative stress in conjunction with mitochondrial dysfunctions (Kerckhove et al., 2017; Staff et al., 2017). Considering all the above, achieving a clear understanding of the pathogenesis of CIPN remains a challenging issue.

## Mitochondria Contribution to CIPN

Recently, mitochondria contribution to painful neuropathy has been extensively reported and defined as an important component in the dysregulation of sensory neurons. For historical review of mitochondria in CIPN in pre-clinical and clinical cases, see Canta et al. (2015) and Flatters (2015). Cumulative evidence has even allowed a drug-based description of mitochondrial dysfunctions as summarized in recent reviews (Canta et al., 2015; Waseem et al., 2018). On a morphological standpoint, atypical mitochondria with swollen matrices and vacuolations have been observed at the clinical level as well as in pre-clinical and *in vitro* models following treatment with paclitaxel, vincristine, cisplatin, oxaliplatin, and bortezomib (Sui et al., 2013; Bennett et al., 2014; Canta et al., 2015; Ma et al., 2018). Assessment of mitochondrial functions revealed alterations in mitochondrial Ca^2+^ homeostasis, deficits in respiration and in ATP production, and induction of apoptotic mitochondrial pathway. All phenomena are indicative of an opening of the mitochondrial Permeability Transition Pore (mPTP), a multiprotein complex located at the contact sites of the mitochondrial outer and inner membranes (MOM and MIM, respectively). By definition, mPTP opening allows the solutes up to ~1.5 kDa to permeate the MIM, which results in equilibration of all small electrolytes across the MIM. These events lead to osmotic swelling of mitochondria, permeabilization of the MOM and disruption of metabolic gradients between the mitochondria and the cytosol. For an extensive review on the history, definition, regulation, and functional consequences of mPTP, see the work by Halestrap (2009). While the exact composition of the mPTP, which initially included the voltage dependent anion channel (VDAC), is still a matter of debate, its opening constitutes a common feature of many pathological conditions (Halestrap and Brenner, 2003) including neurodegenerative diseases (Du and Yan, 2010; Quintanilla et al., 2017).

It is important to mention that MOM permeability to metabolites and ions essentially relies on the presence of VDAC. Considered the most abundant pore-forming proteins in the MOM, VDAC shows both ion selectivity and voltage dependence defining an open (permeable to organic anions ATP, ADP, and to metabolites) and closed (cation selective) states (Hodge and Colombini, 1997; Colombini, 2016). In mammals, three distinct genes encode for three VDAC isoforms (VDAC1, VDAC2, and VDAC3), which display different roles in physiological and pathological conditions, as well as different expression level and tissue specificity (Messina et al., 2012). Beyond their metabolic functions, VDACs also constitute docking sites for several cytosolic proteins, including those involved in apoptosis, energy production, cytoskeletal organization, steroidogenesis, and neurodegeneration [for an exhaustive list, see Caterino et al. (2017)]. In such respect, VDAC functions are closely related to the maintenance of homeostasis and pathological conditions. To the best of our knowledge since the relative distribution of each individual isoform of VDAC has not been determined in human peripheral sensory neurons, the next sections will refer to VDAC without isoform specification.

Following the observation that mitochondrial deficits are apparent before the emergence of pain and nerve terminal degeneration, a “mitotoxicity hypothesis” has been formulated, which establishes a causative link between mitochondrial bioenergetics deficits and abnormal sensitization of distal nerve fibers (Bennett et al., 2011, 2014). In that instance, protection of mitochondrial function has been suggested as a promising therapeutic strategy to alleviate or prevent chronic pain and CIPN. Thus, several mitochondria-targeted peptides or compounds have been developed and assayed in pre-clinical models of CIPN (Flatters et al., 2006; Jin et al., 2008; Melli et al., 2008; Xiao et al., 2009; Toyama et al., 2014, 2018; Areti et al., 2017). The mitigated success encountered so far call for a better understanding of the molecular details and mitochondrial targets at play. Here, we propose to focus on a prevailing hypothesis regarding how a specific class of chemotherapy agents, the microtubule-targeting agents (MTAs), would interfere with mitochondrial function in the context of CIPN. Despite their established potency in the clinics as chemotherapeutic agents, the Vinca-alkaloid drug vincristine and the Taxane drug paclitaxel are the most neurotoxic MTAs. This ever-growing family of compounds has been classified into microtubule-stabilizing agents (MSAs) and microtubule-destabilizing agents (MDAs) based on their effects on the microtubule cytoskeleton at high concentrations (in the 100 nM to micromolar range). In such conditions, they either promote microtubule assembly (MSAs) or trigger microtubule disassembly (MDAs) into tubulin dimer subunits and small oligomers. However, at low and clinically relevant concentrations (low nanomolar range), both MSAs and MDAs primarily suppress microtubule dynamics without significantly affecting the microtubule polymer mass (Dumontet and Jordan, 2010). It is now increasingly accepted that MTAs clinical efficiency cannot solely be the fact of an anti-mitotic activity, as illustrated by the long doubling times of some solid tumors (Komlodi-Pasztor et al., 2011), but rather involve interference with interphase microtubule functions (Mitchison, 2012; Field et al., 2014; Markowitz et al., 2017). By hampering critical cell signaling pathways and preventing microtubules from properly interacting with focal adhesions and adherens junctions (Ogden et al., 2014), MTAs exert complementary anti-angiogenic, anti-vascular, and anti-metastatic properties and proved to be an efficient anti-cancer strategy (Braguer et al., 2008; Schwartz, 2009). Besides their well-known effects on the microtubule network, MTAs also activate the intrinsic apoptotic pathway through direct and indirect actions with mitochondria (Estève et al., 2007). It has notably been shown that MTAs prevent mPTP closure leading to MOM permeabilization and subsequent release of cytochrome c from isolated mitochondria (Evtodienko et al., 1996; André et al., 2000; Varbiro et al., 2001). In other words, MTAs anti-cancer efficiency could be explained by the presence of possibly direct targets on mitochondria. As for the molecular mechanisms underlying MTAs neurotoxic effects, it is generally considered that the same mechanisms are at play in peripheral neurons: direct and indirect targeting of both microtubule/tubulin and mitochondrial networks, as supported by a few studies (Rovini et al., 2010; Shemesh and Spira, 2010; Benbow et al., 2016; Smith et al., 2016). Yet refinements and in-depth investigations of patients and cellular models are needed to provide a clear understanding of CIPN etiology.

The hypothesis we want to discuss here is related to mitochondrial abnormalities observed in neurons from patients and in cellular models of CIPN, following paclitaxel and vincristine treatment. This hypothesis proposes that MTAs target and damage mitochondria by binding to the β-tubulin subunit associated with VDAC, which subsequently prevents mPTP closure. While it is oftentimes referred to such putative mechanism to explain MTAs neurotoxicity (Mironov et al., 2005; Bordet and Pruss, 2009; Zheng et al., 2011; Gornstein and Schwarz, 2014; Nieto et al., 2014; Canta et al., 2015; Brewer et al., 2016; Smith et al., 2016), it is still lacking experimental evidence.

## Scientific Context of the Hypothesis: The Emergence of a New Function for Free Tubulin Dimers

It is the initial finding by Carre of an interaction between tubulin and VDAC that paved the way for the presently discussed hypothesis (Carre, 2002). In this study, the authors first detected the presence of tubulin in mitochondrial fractions isolated from various cancer cell lines. This observation confirmed previous studies reporting tubulin association with mitochondrial membranes in both purified organelles and whole cells (Bernier-Valentin et al., 1983; Saetersdal et al., 1990; Cicchillitti et al., 2008). Moreover, tubulin presence on mitochondrial membranes nicely corroborated the pre-supposed existence of a regulatory element [“Factor-X”, (Saks et al., 1995)] associated with the cytoskeleton to control MOM permeability for respiratory substrates in different muscle types (Kuznetsov et al., 1996). In a recent review, Puurand et al. gathered evidence from all cellular models in which tubulin acts as a regulator of mitochondrial respiration by reducing VDAC permeability to ATP, ADP, and other metabolic substrates across MOM (Puurand et al., 2019). Importantly, the authors highlight how molecular details (i.e., tubulin isotypes) and functional consequences of such regulation happen to be tissue dependent. In the context of this communication, it is relevant to point out the studies from Maldonado and coworkers dedicated to understanding the molecular basis underlying the partial suppression of mitochondrial metabolism that characterizes tumors with a Warburg phenotype (Maldonado et al., 2010, 2013). According to the definition of the Warburg effect, which is a hallmark of cancer (Hanahan and Weinberg, 2011), the malignant cells prefer to produce ATP *via* glycolysis instead of oxidative phosphorylations (OXPHOs) regardless of oxygen supply. In studies using hepatocarcinoma cells as a model, Maldonado and coworkers assessed the hypothesis that closure of VDAC could account for global mitochondrial suppression (Lemasters and Holmuhamedov, 2006). The authors first established that free cytosolic tubulin dynamically modulates mitochondrial membrane potential (Maldonado et al., 2010) and proposed VDAC to be the target of free tubulin based on the earlier findings by Rostovtseva and coworkers that nanomolar concentrations of dimeric tubulin close VDAC reconstituted into planar lipid bilayers and suppresses respiration of isolated mitochondria and permeabilized cells (Monge et al., 2008; Rostovtseva et al., 2008; Timohhina et al., 2009). Subsequent VDAC knockdowns in hepatocarcinoma cells confirmed (1) the isoform-dependent relevance of VDAC in mitochondrial membrane potential formation and (2) the role of tubulin, which by closing VDAC acts as a brake to suppress mitochondrial metabolism and therefore contributes to the Warburg effect. It is necessary to point out that a direct assessment of VDAC opening and closure in a cellular context is hardly achievable, leaving uncertainties about the actual status of the channel and its dynamic changes. Nevertheless, significant insights about tubulin interaction with VDAC were obtained from VDAC reconstituted into planar lipid membranes. Notably, the demonstration that the tubulin-blocked state of VDAC controls the channel selectivity for cations and permeability to ATP (Gurnev et al., 2011). In addition, parameters such as the length and charges of tubulin C-terminal sequences, membrane lipid composition, and phosphorylation state of the channel were shown to be crucial in the regulation of tubulin interaction with VDAC (Rostovtseva et al., 2008, 2012, 2017, 2018; Gurnev et al., 2012).

In the scientific context of the study from Carre, the physiological relevance of tubulin interaction with VDAC has been connected with VDAC participation to mPTP and MOM permeabilization during apoptosis. Since MTAs could directly induce the mPTP opening and the release of cytochrome *c* from isolated mitochondria (André et al., 2000), tubulin associated with VDAC could constitute an appropriate target for MTAs to trigger apoptosis. Subsequent genetic investigations have found VDAC to be dispensable in mitochondria-mediated cell death associated with mPTP opening (McCommis and Baines, 2012; Bernardi, 2013; Baines and Gutiérrez-Aguilar, 2018). However, McCommis and Baines recently exposed and discussed alternative models for VDAC’s role in MOM permeabilization and induction of apoptosis independently of the mPTP (McCommis and Baines, 2012). One of these models involves binding of pro-apoptotic partners to VDAC inducing the channel closure, which would then result in an accumulation of mitochondrial metabolites and ultimately outer membrane permeabilization by a yet undefined mechanism. Nonetheless, it is still not demonstrated whether tubulin would fit this model and act similarly in the context of MTA-induced apoptosis.

## Moving Further: New Insights on the Components of a Still Untested Hypothesis

To refine and critically re-evaluate the hypothesis in the specific context of MTA-induced CIPN, it would be essential to understand the molecular details related to the different components involved. In particular, we address in the following sections questions about MTA-dimeric tubulin complex and its interaction with VDAC and the potential relevance of both tubulin isotypes and the free versus polymerized ratio of tubulin in relation to the hypothesis.

## The MTA-Dimeric Tubulin Complex and its Interaction with VDAC

Tubulin associated with mitochondrial membranes was the first candidate proposed to explain direct effect of MTAs, as anti-cancer agents, on mitochondria isolated from cancer cell lines. This implies that MTAs access the tubulin dimer bound to mitochondrial membrane, raising immediate question about tubulin dimer orientation at the membrane surface and identification of its membrane-binding domain. Recently, by combining several biophysical methods, Hoogerheide et al. identified α-tubulin’s amphipathic helix H10 as responsible for dimeric tubulin binding to biomimetic “mitochondrial” membranes (Hoogerheide et al., 2017). Importantly, according to these data, MTAs binding sites on the β-subunit remain accessible, as suggested by Bhattacharyya and Wolff, who showed that vinblastine-binding affinity to β-tubulin was unchanged between membrane bound and solubilized tubulin (Bhattacharyya and Wolff, 1975). The initial statement of the hypothesis (Carre, 2002) introduced the notion that MTAs could alter tubulin dimer conformation in such a way that it would lead to mPTP opening. In spite of the paucity of data in this regard, atomic models based on electron crystallographic density and molecular dynamics simulation of the bioactive conformation of MTAs bound to soluble tubulin heterodimer show that both Taxol® and Vinca-alkaloids induce substantial conformational changes in the tubulin dimer structure (Nogales et al., 1998; Snyder et al., 2001; Xiao et al., 2006; Mitra and Sept, 2008). These changes primarily affect the assembly properties of the heterodimer into microtubules (Lobert and Correia, 2000; Coderch et al., 2012). How and whether MTAs bound to soluble tubulin heterodimer would impact tubulin dimer interaction with mitochondrial membranes or with VDAC remains unanswered. On a different standpoint, some MTAs such as colchicine and Taxol® have been reported to alter both artificial and biological membrane physical properties (i.e., phospholipid phase transitions, lipid order parameters, fluidity) (Balasubramanian and Straubinger, 1994; Mons et al., 2000; Ashrafuzzaman et al., 2012), which might be considered as another mechanism of MTA-induced disruption of tubulin/VDAC interaction.

## Nature of the Tubulin Isotype Interacting with VDAC at the MOM

In humans, at least seven α-tubulin and eight β-tubulin genes encode for tubulin isotypes, accounting for the remarkable variety of heterodimeric combinations found with respect to the cell type and stage of development (Ludueña, 2013; Roll-Mecak, 2019). Only a few studies have characterized the tubulin associated with mitochondrial fractions. Carre has detected α-tubulin and classes I, II, III, IV β-tubulin, in variable proportions (Carre, 2002). They also noticed a marked accumulation of the βIII-tubulin isotype in the neuroblastoma cell line SK-N-SH. Subsequent studies have confirmed the finding of β-tubulin isoforms associated with mitochondrial fractions, specifically of βII-tubulin in mitochondria samples isolated from rat cardiomyocytes (Guzun et al., 2011) and of βIII-tubulin in ovarian cancer cells (Cicchillitti et al., 2008). Interestingly, using a combination of two-dimensional gel electrophoretic profiles and chromatography affinity assays, Cicchillitti et al. characterized a cytoskeletal and a mitochondrial class III β-tubulin. According to the authors, one isoform showed specific post-translational modifications (glycosylation and phosphorylation) and was compartmentalized into the cytoskeleton, while the other isoform, unglycosylated and unphosphorylated, was instead found exclusively localized to mitochondria. Together with the report by Carre (2002) of a ~3-time increase of βIII-tubulin in mitochondrial fractions compared to the whole cell, such pattern of intracellular distribution might be indicative of a regulatory role of βIII-tubulin at the mitochondrial level. It is worth mentioning the particular interest in the cancer research field attributed to βIII-tubulin, since it has been associated with tumor development, aggressiveness, as well as resistance to chemotherapy – notably to MTAs – in tumors with poor prognosis (Seve et al., 2005; Katsetos et al., 2011). A proposed chemoresistance mechanism in tumors with increased levels of βIII-tubulin expression (Derry et al., 1997; Gan et al., 2011) involves the finding that Taxol® has a lesser binding affinity for βIII compared to other β-tubulin isotypes (Yang and Horwitz, 2017). Further analysis of the β-tubulin isotype sequences near the Taxol® binding site specifically identified an alanine residue (Ala218) in βIII, where other isotypes contain a threonine (Yang et al., 2016). Applying molecular dynamic simulations, this one residue change was sufficient to result in a significant decrease in the drug binding to this isotype compared with other β-tubulins. Therefore, the overexpression of βIII isotype could limit the binding of Taxol®, which would account for its reduced clinical potency in such type of tumors.

## Free/Polymerized Tubulin Ratio in the Context of CIPN

Sensory and motor neurons that compose the peripheral nervous system rely on ATP produced by OxPhos to maintain proper functioning and sustain their high energy requirements (Baloh, 2008). Mitochondrial energetic failure and altered mitochondrial transport in distal axons are commonly observed in neurodegenerative disorders with axonal degeneration, including in CIPN. Would it be conceivable that tubulin regulates the mitochondrial bioenergetics of sensory neurons? Would MTAs affect tubulin availability in affected neurons in such a way that it would alter tubulin regulation of mitochondria permeability for adenine nucleotides and metabolite substrates?

The extent of the microtubule damages reported the following MTA treatments in animal and cellular models of CIPN seems to be quite variable. For instance, initial animal studies described a significant decrease in microtubule density in sensory neurons from rat models of vincristine-induced peripheral neuropathy (Tanner et al., 1998; Topp et al., 2000). It is worth mentioning that these studies employed different MTAs injection modes and administrated concentrations in which clinical relevance has been debated (Nakata and Yorifuji, 1999; Theiss and Meller, 2000; Gornstein and Schwarz, 2014). By contrast, recent studies employing cellular models of MTA-induced neurotoxicity have revealed alterations in microtubule dynamics parameters (Rovini et al., 2010; Shemesh and Spira, 2010) or tubulin biochemistry (Benbow et al., 2016; Cook et al., 2018; Wozniak et al., 2018), rather than massive ultrastructural changes of the microtubule network. Therefore, alteration of tubulin regulation of mitochondrial permeability to metabolic substrates remains to be explored as a possible mechanism by which MTAs would disrupt neuronal energy homeostasis.

## Conclusion

The search for efficient preventive and curative treatment options against CIPN constitutes primary challenges in current oncology practice. Many aspects of the physiology, especially at the molecular level, have not been resolved. Here, we tried to dissect one of the suggested mechanistic hypotheses, which involve the major metabolite channel of the outer membrane VDAC and its regulation by dimeric tubulin in the etiology of CIPN following MTA treatments. While the foundations of this hypothesis provide an explanation for mitochondrial apoptosis triggered by MTAs in cancer cells, its relevance as a neurotoxic mechanism requires further assessment. Since the first mention of the interaction between dimeric tubulin and VDAC as a putative target of MTAs in neurons, significant knowledge has been gained and tubulin is now considered as a regulatory component of VDAC permeability to adenine nucleotides and ultimately of mitochondrial function in different types of tissues. Similarly, for years, studies of VDAC have confined the importance of this channel to cancer cells as a modulator of mitochondrial membrane permeability and ultimately of apoptosis induction. In the specific context of CIPN, VDAC possible involvement in mitochondrial dysfunction has never been particularly addressed except as a structural component of the mPTP (Canta et al., 2015; Brewer et al., 2016; Waseem et al., 2018). Since the current state of knowledge dissociates the MOM channel from the mPTP, VDAC contribution to mitochondrial damages associated with CIPN might be thought from a different perspective. Lately, the interest for this channel has spanned into the neuroscience field with the successive findings of VDAC1 serving as the docking site for several neurodegenerative disease-related misfolded proteins (Magri and Messina, 2018). As a result, VDAC is considered as a potent pharmacological target for new molecules and peptides (Magri and Messina, 2018), which potentially protects against mitochondrial dysfunctions associated with neurodegenerative disorders. A multidisciplinary approach to further clarify the relevance of tubulin regulation of mitochondrial bioenergetics in neurons would certainly benefit to CIPN field of research. Unraveling the molecular basis of MTA-induced neurotoxicity in the peripheral nervous system may contribute as well to understanding the complex interplays between the cytoskeleton and the mitochondrial energy metabolism.

## Data Availability

All datasets generated for this study are included in the manuscript and/or the supplementary files.

## Author Contributions

The author confirms being the sole contributor of this work and has approved it for publication.

### Conflict of Interest Statement

The author declares that the research was conducted in the absence of any commercial or financial relationships that could be construed as a potential conflict of interest.

## Abbreviations

ADP: Adenosine diphosphate
ATP: Adenosine triphosphate
CIPN: Chemotherapy-induced peripheral neuropathy
CTT: C-terminal tail
MDA: Microtubule-destabilizing agent
MIM: Mitochondrial inner membrane
MOM: Mitochondrial outer membrane
mPTP: Mitochondrial permeability transition pore
MSA: Microtubule-stabilizing agent
MTA: Microtubule-targeting agent
OxPhos: Oxidative phosphorylation
VDAC: Voltage-dependent anion channel.
